# Gaussian Process Based Bayesian Inference System for Intelligent Surface Measurement

**DOI:** 10.3390/s18114069

**Published:** 2018-11-21

**Authors:** Ming Jun Ren, Chi Fai Cheung, Gao Bo Xiao

**Affiliations:** 1 State Key Laboratory of Mechanical System and Vibration, School of Mechanical Engineering, Shanghai Jiao Tong University, Shanghai 200245, China; renmj@sjtu.edu.cn; 2 State Key Laboratory of Ultra-Precision Machining Technology, The Hong Kong Polytechnic University, Hong Kong, China; mfbenny@inet.polyu.edu.hk

**Keywords:** Surface measurement, multi-sensor measurement, surface modelling, data fusion, Gaussian process

## Abstract

This paper presents a Gaussian process based Bayesian inference system for the realization of intelligent surface measurement on multi-sensor instruments. The system considers the surface measurement as a time series data collection process, and the Gaussian process is used as mathematical foundation to establish an inferring plausible model to aid the measurement process via multi-feature classification and multi-dataset regression. Multi-feature classification extracts and classifies the geometric features of the measured surfaces at different scales to design an appropriate composite covariance kernel and corresponding initial sampling strategy. Multi-dataset regression takes the designed covariance kernel as input to fuse the multi-sensor measured datasets with Gaussian process model, which is further used to adaptively refine the initial sampling strategy by taking the credibility of the fused model as the critical sampling criteria. Hence, intelligent sampling can be realized with consecutive learning process with full Bayesian treatment. The statistical nature of the Gaussian process model combined with various powerful covariance kernel functions offer the system great flexibility for different kinds of complex surfaces.

## 1. Introduction

Surface size, geometry and texture are some of the most influential subjects in the field of precision engineering [1]. The development of advanced machining technologies allows vast application of complex surfaces superimposing multiple scales of feature in mechanical and optical engineering for their superior performance in terms size reduction and versatile functions [1,2,3]. There is a growing awareness of the importance of these new types of surfaces in modern science and technologies [4]. To ensure the functionality of the components, these surfaces are required to be fabricated with high precision in terms of form accuracy in sub-micron range and surface finishing at nanometric level. However, the geometric complexity of these advanced surfaces requires multi-scale measurement and characterization, which imposes a lot challenges for current precision surface metrology [5,6].

Extensive research has been conducted on developing various measurement instruments to fulfill a wide range of metrological needs [7,8,9,10], such as high precision coordinate measuring machines [7,8], micro topographical instruments [11], electron microscopy [12], optical interferometry [13,14,15], etc. Although these instruments are capable of performing accurate and efficient measurement at specific measurement range, few of them could realize high dynamic range multi-scale measurement with high efficiency and accuracy. Integrating several complementary sensors into an instrument therefore becomes a promising solution to address complicated measurement tasks. For instance, integrating tactile or scanning probe with imaging sensors is an attractive solution for precision measurement of large scale complex surfaces with high efficiency [16,17]. However, multi-sensor measurement also brings many challenges, such as more sophisticated measurement strategy, effective multi-sensor data fusion, and complex uncertainty propagation. The effectiveness of multi-sensor measurement largely depends on appropriate measurement strategy so as to guide the process to perform cooperative measurement [5]. Model-based methods have been considered as the effective solution for multi-sensor measurement [18,19,20,21]. Unlike the conventional sampling strategy which is normally designed prior the actual measurement, the model-based methods consider the measurement as a time-series optimization process such that sampling plan is adaptively determined based on the model which are established using prior measured dataset in each iteration. Obviously, the surface modelling forms the beating heart of the model-based sampling strategy. Although there are a large amount of literature on this topic [22,23,24], the surface modelling in multi-sensor system is still a challenging task since the measured datasets are normally embedded in different coordinate frames with different resolutions and different levels of uncertainties. This requires the surface modelling methods be capable of efficiently fusing multi-sensor measured datasets and hopefully be capable of performing self-assessment so as to give some hints to the sample distribution [25]. 

Considering the characteristics of the multi-sensor measurement, the modelling process can generally be divided into two steps, including data registration and fusion [26]. In the first step, data registration algorithm is used to register all the measured dataset to a common coordinate frame via matching process. Although there are several mature data registration algorithms, such as iterative closest point algorithm [27], the results would inevitably include systematic errors due to the existence of measurement errors, which is seldom considered in the measurement of complex surfaces. In the second step, the overlapped datasets are fused to construct a unique surface model. Advanced fusion algorithm should be capable of using the redundant data to deduce a fused result with reduced measurement uncertainty. Data fusion have widely been studied in fields like signal processing, computer vision, and control, and some of the fusion techniques have started to be used in coordinate metrology. A review of current data fusion in coordinate metrology have been presented by Wang et al. and categorized the existing methods into four groups, including repeated measurements, stitching, range image fusion, and 3D data fusion [28]. Repeated measurement and stitching techniques are widely used in optical interferometry for enlarging the measurement range. The fusion is relatively simple since the datasets are obtained from same sensors and should have save level of uncertainties. Range image and 3D data fusion is however difficult, which normally involves six degree of registration and sophisticated data fusion process. Several data fusion methods have been presented, such as weighted least square based fusion and scale-decomposition based fusion [28]. However, these methods are either suspicious to the geometry being modelled or difficult to perform self-assessment of the fused result, which makes it ineffective to be served as a modelling algorithms to perform automatic and intelligent sampling of the surface during the measurement. 

Recently, Gaussian process (GP) has been used to address the multi-sensor data fusion problem [29]. GP is kind of a non-parametric Bayesian inference model which has been proved a powerful mathematical tool to model various complex surfaces in terms of spatial covariance functions [30,31,32]. The statistic nature of GP makes it capable of incorporating the measurement errors into the modelling process and assigning credibility to the constructed model. Taking these advantages, this paper presents a GP based Bayesian inference system for the realization of intelligent surface measurement on multi-sensor system. The system is responsible for multi-feature classification and multi-dataset regression. Multi-feature classification is capable of performing automatic extraction and classification of the features of the measured surfaces so as to design appropriate covariance kernel functions and corresponding initial sampling strategy. Multi-dataset regression is capable of optimizing the sampling strategy via multi-sensor data fusion and on-line sampling adaptation by taking the uncertainty of the Gaussian process model as critical sampling criteria. Hence, automatic measurement setup and intelligent sampling strategy can be realized with consecutive learning process with full Bayesian treatment. Experiments are conducted to verify the effectiveness of the proposed study.

The remaining sections are outlined as follows. Section 2 states the idea that how a surface measurement can be treated as a regression problem by taking GP as mathematical foundation. The GP-based Bayesian inference system is then presented in Section 3 with detailed introduction of the two main parts of the inference system, i.e., the multi-feature classification and multi-dataset regression. Experimental work is given in Section 4 to verify the effectiveness of the proposed method, and a conclusion is made in Section 5.

## 2. Preliminary

Due to the limited resolution and the error of a measurement instrument, surface measurement can generally be formulated as a regression problem of the form, where S(x) and $\varepsilon_{x}$ are the measured data and the associated measurement error at x, f(x) is the evaluated surface model which is used to give prediction to the unmeasured area of the surface. Since f(x) is generally an unknown function for a manufactured surface, its prediction is inevitably uncertain. Considering the statistic nature of the surface measurement, Gaussian process (GP) [31], a non-parametric Bayesian inference model, is used to address this problem.

GP is basically a collection of random variables, any finite subset of which obeys joint Gaussian distribution. It is generally be defined by a mean function μ(X) and a covariance matrix K(X,X) [31]

(1) GP(X)~N(μ(X),K(X,X))

where $X=\left[x_{1},x_{2},\ldots ,x_{n}\right]$ is the locations of the measured data S. In present study, the mean function is set to be zero-offset by scaling the data appropriately such that it has an empirical mean of zero. The covariance matrix K(X,X) is generally formulated by covariance kernel function $k\left(x_{i},x_{j}\right)$ in the form as given by Equation (2) where $\left(x_{i},x_{j}\right)$ are the locations of arbitrary two points on the GP model.

(2) $$K(X,X)=[\begin{array}{ccccc} k(x_{1},x_{1}) & k(x_{1},x_{2}) & k(x_{1},x_{3}) & \cdots & k(x_{1},x_{n})\\ k(x_{2},x_{1}) & k(x_{2},x_{2}) & k(x_{2},x_{3}) & \cdots & k(x_{2},x_{n})\\ k(x_{3},x_{1}) & k(x_{3},x_{2}) & k(x_{3},x_{3}) & \cdots & k(x_{3},x_{n})\\ \vdots & \vdots & \vdots & \ddots & \vdots \\ k(x_{n},x_{1}) & k(x_{n},x_{2}) & k(x_{n},x_{3}) & \cdots & k(x_{n},x_{n}) \end{array}]$$

Hence, according to the Bayesian theory, a prediction of $f^{*}$ at an arbitrary location $x^{*}$ on unmeasured region of the model can be evaluated by the joint distribution of $f^{*}$ with measured data S as follows:

(3) $$\left[\begin{array}{c} S\\ f^{*} \end{array}\right]\sim N\left(\begin{array}{cc} 0, & \left[\begin{array}{cc} K\left(X,X\right)+\sigma_{\varepsilon }^{2}I & K\left(X,x^{*}\right)\\ K\left(X,x^{*}\right) & K\left(x^{*},x^{*}\right) \end{array}\right] \end{array}\right)$$

where I is the identity matrix; $\sigma_{\varepsilon }$ is a hyperparameter representing the variance of the random part of the measurement error. The covariance cov and the mean $m^{*}$ of the $f^{*}$ can then be obtained by the marginal distribution of $f^{*}$ as given by Equations (4) and (5).

(4) $$m^{*}=K\left(x^{*},X\right){\left(K\left(X,X\right)+\sigma_{\varepsilon }^{2}I\right)}^{-1}Z$$

(5) $$\mathrm{cov}\left(f^{*}\right)=K\left(x^{*},x^{*}\right)-K\left(x^{*},X\right){\left(K\left(X,X\right)+\sigma_{\varepsilon }^{2}I\right)}^{-1}K\left(X,x^{*}\right)$$

**Figure 1:**
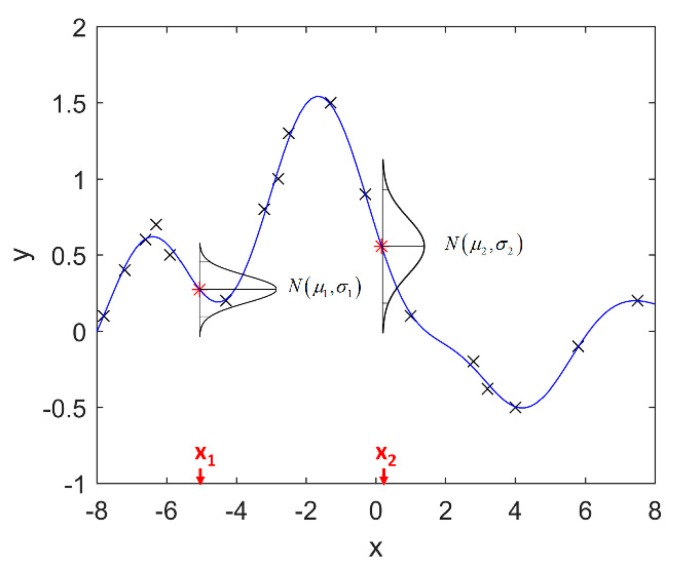
An example of GP modelling and prediction.

It is seen that the GP inference is determined by the covariance forms which represents the covariance of the points on the model. Hence the construction of appropriate covariance kernel function is the core of GP modelling. Figure 1 shows an example of GP modelling and prediction. The crosses are the measured points, in which x is the locations and y is the corresponding measured value. The line is the established GP model by taking the measured point as training data. Two predictions are made at unmeasured location x1 and x2. $\mu_{1}$ and $\mu_{2}$ are the predictions, and the $\sigma_{1}$ and $\sigma_{2}$ are their evaluated uncertainties. 

## 3. Gaussian Process Based Bayesian Inference System

### 3.1. System Configuration

**Figure 2:**
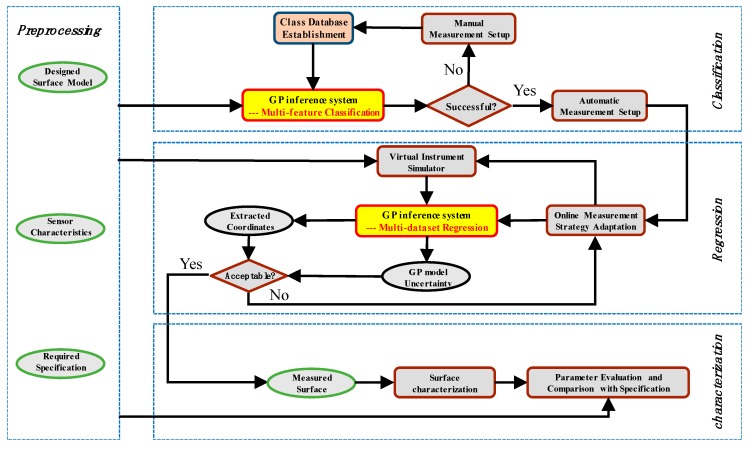
Schematic diagram of GP-BIS.

Figure 2 shows the schematic diagram of the GP based Bayesian inference system (GP-BIS), which is mainly composed of four modules, including preprocessing, multi-feature classification, multi-dataset regression, and surface characterization. In preprocessing, the characteristics of the involved measurement sensors are analyzed and calibrated, including measurement range, resolution, speed, errors, etc. The designed models of the measured surfaces are transformed to a unified format and the properties of the measured parts such as hard and soft materials, and the required specifications, e.g., form accuracy and roughness, are loaded. 

Taking the information of the preprocessing as input, multi-feature classification module extracts and classifies the geometric features of the designed models so as to design an appropriate covariance kernel functions for GP modelling. An initial measurement strategy is also loaded from a pre-established class database. The class database is responsible for collecting appropriate class of measurement strategies for specific tasks with respect to certain types of surface features based on the prior knowledge. If the contained surface features and the required specifications are successfully classified, the measurement strategy will be automatically loaded. Otherwise, manual design of the sampling strategy is required and a new class will be uploaded to the database so that they can be successfully classified in future. A certain class in the database would contain necessary information for the setup of the measurement instruments, including the types of the sensors and the associated parameters which will be used during measurement. For contact types of sensors, such as trigger probe, the initial sampling patterns may be also included for further optimization in GP modelling process. It is emphasized that the sampling strategies produced in this stage are only initial plans which require further optimization. Multi-dataset regression module is responsible further optimizing the initial measurement strategy. This is carried in two steps. Firstly, taking the designed covariance kernel function as input, GP model is established to fuse the datasets obtained from different sensors to obtain a unique measured surface. Secondly, both the bias and the uncertainty of the fused surface model is used to qualify the reliability of the measured surface, and is served as critical sampling criteria to perform adaptive sampling until reaching desired reliability. During the process, a virtual instrument simulator [33] can also be developed to analyze the error distribution of each measured data by taking the sensor characteristics and the adopted sampling strategy as input. The analyzed error distribution of each measured data can be used to aid the surface modelling and uncertainty analysis. At the end, the accepted surface model is then used to perform scale-decomposition, feature segmentation, or pattern analysis to fully characterize the quality of the machined surface in different scales [2].

### 3.2. Design of Covariance Kernel via Multi-Feature Classification

Covariance kernel is a function used to describe the spatial correlation of a geometric feature in GP modelling, and hence the design of a covariance kernel should be in accordance to the characteristics of the measured surface. For a machined surface with known CAD model, the characteristics of the surface topography, such as smoothness, roughness, and periodicity can be specified and classified easily. In GP-BIS, such knowledge is the key to design covariance kernel functions and would significantly improve the measurement efficiency and accuracy. There already exists various covariance kernel functions which possess variety of geometric characteristics [31,34]. Table 1 summarizes currently widely used kernel functions and their characteristics, including white noise (WN), linear (LIN), square exponential (SE), periodic (PER), Matérn class (MC), rational quadratic (RQ), neural network (NN), and piecewise polynomial (PP). Different kernel functions can be selected to study different kinds of surfaces in accordance with the characteristics. Currently, the basic kernel functions are pre-assigned to different types of topographies based on the characteristics as listed in Table 1. For instance, SE is infinitely differentiable and hence is suitable in studying smooth surface, while PER would be a good choice for the surfaces possessing periodic features. 

**Figure 3:**
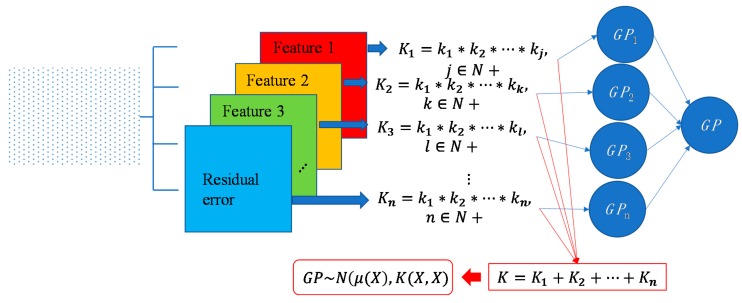
Design of composite covariance kernel functions.

For some surfaces containing multiple features at different scales, a single covariance kernel may not be flexible enough to describe the spatial correlation. In such case, the listed kernel functions can be served as base functions to form various composite kernel functions by combining them together. Figure 3 shows the design of the composite kernel functions. It starts from decomposing the geometric feature of a surface in different scales. Appropriate kernels are selected for the decomposed features in each scale and then a composite kernel function can be formed by simply combining the selected kernels. For instance, if a smooth surface is superimposed by periodic micro-structures, a combination of SE and PER can be designed to model two types of features simultaneously. For some scenario, even the decomposed feature in a scale may be complex that cannot be appropriately described by a single kernel. In such case, besides summation, multiplication among the kernels can also be performed to further enhance the flexibility of the model. Kernel summation can be used to simultaneously model different types of spatial correlation while kernel multiplication can be used enhance the flexibility of a kernel for modelling a complex spatial correlation. The fitness of the designed kernel function should well balance the model fitness and complexity which can be qualified by the Bayesian information criterion [34]. This offers GP a great flexibility in modelling different types of complex surfaces by combining kernel summation and multiplication. 

### 3.3. Sampling Strategy Adaptation via Multi-Dataset Regression

One of the important technical merits of multi-sensor measurement is capability in improving the quality of the measurement results via data fusion process. Considering that all the measured datasets are from a common surface, they should be inherently correlated. Hence, besides the spatial correlation within each dataset, the correlation across the datasets should also be taken into account. In GP modelling, this can be formulated by modelling the auto-covariances and cross-covariances among the measured datasets [34]. For arbitrary two given dataset, the mean and the covariance can be given by Equations (6) and (7).

(6) $$m^{*}=\left[K\left(x^{*},X_{1}\right),K\left(x^{*},X_{2}\right)\right]K\left(X_{12},X_{12}\right){\left[Z_{1},Z_{2}\right]}^{T}$$

(7) $$\mathrm{cov}\left(f^{*}\right)=K\left(x^{*},x^{*}\right)-\left[K\left(x^{*},X_{1}\right),K\left(x^{*},X_{2}\right)\right]K{\left(X_{12},X_{12}\right)}^{-1}\left[K\left(X_{1},x^{*}\right),K\left(X_{2},x^{*}\right)\right]$$

where

(8) $$K\left(X_{12},X_{12}\right)=\left[\begin{array}{cc} K\left(X_{1},X_{1}\right)+\sigma_{\varepsilon 1}^{2}I & K\left(X_{1},X_{2}\right)\\ K\left(X_{1},X_{2}\right) & K\left(X_{2},X_{2}\right)+\sigma_{\varepsilon 2}^{2}I \end{array}\right]$$

$X_{1}$ and $X_{2}$ are the locations of the measured datasets $Z_{1}$ and $Z_{2}$ from two different sensors, $K\left(X_{1},X_{1}\right)$ and $K\left(X_{2},X_{2}\right)$ are the auto-covariances of $X_{1}$ and $X_{2}$ respectively, $K\left(X_{1},X_{2}\right)$ is the cross-covariance between the $X_{1}$ and $X_{2}$, $\sigma_{\varepsilon 1}$ and $\sigma_{\varepsilon 2}$ are the hyperparameters representing the variance of the random part of the $X_{1}$ and $X_{2}$ respectively. 

One of immediate advantage of above multi-dataset regression is that it preserves the full Bayesian inference during the fusion process so that credibility can still be assigned to the fused model using the covariance of the established GP model as given in Equation (7). Hence, the measurement process can generally be considered as a time-series learning process that the credibility of the fused model can be served as critical sampling criterion to perform on-line sampling adaptation. As shown in Figure 2, it starts from extracting a set of data from the surface using the initial sampling strategy obtained in multi-feature classification module, and multi-dataset regression is then performed to establish a fused GP from the measured datasets and evaluate the covariance of the model. If the covariance exceeds a pre-specified threshold, it is considered that the sampling is insufficient to lean the surface geometry accurately and more data are sampled at the regions where the maximum covariance is located. By such a consecutive learning and adaptive sampling process, a measured surface model with acceptable reliability can be obtained efficiently. 

## 4. Experimental Study

### 4.1. Computer Simulation on Surface Modelling

The proposed GP-BIS has been implemented in MATLAB 2018a (from The MathWorks, Natick, USA) and tested on several surfaces with variety of complexities. Firstly, case studies have been conducted on multiple features enriched complex surfaces to study the capability of the GP modelling. A set of 121 × 121 grid points are extracted from the designed surface in each case study, and are added Gaussian noise with 2 μm standard deviation to simulate the measurement error. Table 2 shows complex surfaces which are made of a parabola and different scales of sinusoidal waves. It starts from a simple parabola given by Equation (10). Since the surface is generally a simple smooth surface, GP-BIS selects the SE as the covariance kernel functions. It is clearly seen from the residual map that the surface has been accurately modelled and the contained random errors has been successfully rejected. This can be explained from the principle of the GP which is basically a process to find a correlation that best explains the spatial distribution of the sampled points. Hence, the uncorrelated part, i.e., the added random errors will be considered as measurement errors, which is incorporated in the modelling process by $\sigma_{\varepsilon }I$ in Equation (3). In experiment, $\sigma_{\varepsilon }$ has been evaluated to be 1.99 μm which is very close to the true value.

(9) $$z=-0.3(0.0625x^{2}+0.0625y^{2})\;x,y\in [-10,10]\;\mathrm{mm}$$

Then, a sinusoidal wave with amplitude 0.4 mm and period 5 mm in both two X and Y directions is superimposed to the parabola to make the surface more complex. The designed surface now has two radically different topographies in two different scales. Hence, in the multi-classification process, the GP-BIS identifies two different types of topographies and a new composite kernel functions has been designed using SE and PER (see Table 1). The measurement component $\sigma_{\varepsilon }$ has been identified to be 2.18 μm and hence the surface has also been accurately modelled. To further improve the complexity of the surface, another sinusoidal wave with amplitude 0.2 mm and period 1 mm in both two X and Y directions is further superimposed to the surface designed in second case study. GP-BIS identifies the third different topographies, and a three level of composite kernel SE + PER + MC has been designed. The measurement error part is identified to be 2.23 μm, which further verifies the accuracy of the established GP model. Intuitively, considering the prior knowledge of three different features, SE + PER + PER seems also a good choice for the surface. The results, i.e., the last row of Table 2 show that SE + PER + PER also achieved reasonably good result while it is still not compatible with SE + PER + MC. This is due to the reason that, after the use of SE + PER, the scale of the remaining feature may be affected by the modelling residual. Hence more flexible MC would be better choice in modelling the remaining part which is a combination of the third scales of feature and the modelling residual of the two larger scales of features.

### 4.2. Actual Application on Multi-Sensor Instrument

The proposed GP-BIS has applied to an instrument equipped with a touch trigger probe and a laser scanner. Trigger probe is one of typical sensors which can achieve high accurate measurement with generally very low efficiency. The laser scanner, on the other hand, is capable of realizing high efficient measurement while with relatively low accuracy. For the measurement of complex surfaces, more intelligent multi-sensor measurement strategy is required to achieve both high accurate and high efficient measurement. The probing error and the linearity of the laser scanner are identified to be 0.9 μm (1σ, normal) and 15.2 μm (uniform). 

Measurement has been conducted on a machined workpiece which is designed by a peak function defined by Equation (10).

(10) $$\begin{array}{l} z=6{\left(1-\frac{x}{16}\right)}^{2}\cdot \exp\left(-{\left(\frac{x}{16}\right)}^{2}-{\left(\frac{y}{16}+1\right)}^{2}\right)-20\left(\frac{x}{5\times 16}-{\left(\frac{x}{16}\right)}^{3}-{\left(\frac{y}{16}\right)}^{5}\right)\cdot \exp\left(-{\left(\frac{x}{16}\right)}^{2}-{\left(\frac{y}{16}\right)}^{2}\right)\\ \;-\frac{2}{3}\exp\left(-{\left(\frac{x}{16}+1\right)}^{2}-{\left(\frac{y}{16}\right)}^{2}\right),\;x,y\in [-40,40] \end{array}$$

**Figure 4:**
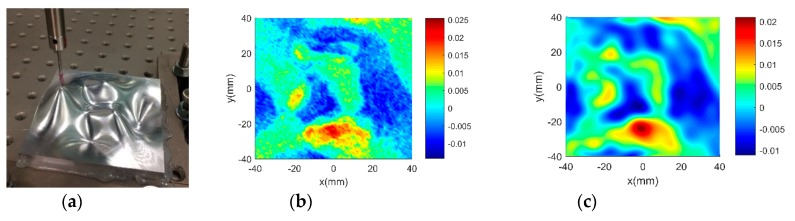
Measurement of a designed complex surfaces on multi-sensor instrument. (**a**) machined workpiece; (**b**) Benchmarking form error; (**c**) multi-sensor fused form error.

Figure 4a shows the machined workpiece. The measurement has been conducted in four steps. In the first step, touch trigger probe is used to extract a set of dense points, i.e., 6456 points with uniform spacing over the entire surface. The results are considered sufficiently accurate and is served as benchmarking to verify other more efficient measurement. Secondly, the laser scanner is used to measure the surface with high density. The measurement is very fast while the accuracy would be low. Thirdly, touch trigger probe is used to perform adaptive sampling based on multi-dataset regression module. Since there is only a single data source, the multi-dataset regression becomes a single GP modelling. It has been noted in published literature [18,32] that GP based adaptive sampling has superior performance than other sampling methods in sampling complex surfaces. Hence, the results are also used as benchmarking to examine the efficiency of the proposed GP-BIS. Fourthly, by taking the measurement result of the laser scanner as input, adaptive sampling is conducted using touch trigger probe. Hence, in the multi-dataset regression, the datasets obtained by the laser scanner and the trigger probe should be fused to establish a GP model in each adaptation. Hence, the GP-BIS becomes producing multi-sensor measurement strategy in the forth experiment. 

Figure 4b,c shows the evaluated results of benchmarking and multi-sensor fused dataset by using best fitting method [35] which is one of the typical methods in form characterization of complex surfaces. It performs the form characterization of the measured dataset by best fitting the dataset to the corresponding designed surface via rigid body transformation, and the error of the dataset can be evaluated by projecting it onto the designed surface. The measured number of points and the root-mean-square (RMS) of the height error are used to characterize the efficiency and accuracy of different measurement methods. A summary of the results is given in Table 3.

Although laser scanner has extremely good measurement efficiency, the accuracy is very low comparing with touch trigger probe. With the aid of the GP-BIS, the trigger probe can perform adaptive sampling, and the efficiency has been dramatically improved and the accuracy has been preserved at the same time. With the aid of the laser scanner, multi-dataset regression is used to further improve the measurement efficiency around 40% comparing with pure triggering measurement. The results demonstrated the capability of the proposed GP-BIS in enhancing the performance of the multi-sensor instrument in measuring complex surfaces.

## 5. Conclusions

This paper presented a GP-BIS for realizing automatic and intelligent surface measurement on multi-sensor instruments. The system considers the surface measurement as a consecutive learning process and makes use of Gaussian process to model the process based on two key modules including multi-feature classification and multi-dataset regression. The system has been implemented and applied to produce multi-sensor measurement strategy for an instrument which is equipped with a trigger probe and a laser scanner. The results shows that the performance of the multi-sensor instrument has been dramatically improved around 40% in terms of measurement efficiency while maintains the measurement accuracy at the save level. The proposed study should provide new insight for the realization of intelligent measurement of complex surfaces. One of the problem of GP modelling is the high computation complexity which would lead huge time for the computation when large number of data is involved in the inference process. Future work considers the implementation of approximated GP inference with the aid of graphics processing unit (GPU) for the practical use of the proposed method especially in manipulating vision based measurement dataset.

## Figures and Tables

**Table 1:** Geometric characteristics of 8 base kernel functions.

	Geometric Characteristic	Base Function	Gp Prior
WN	White noise	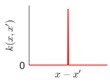	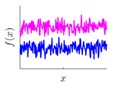
LIN	linearly varying amplitude	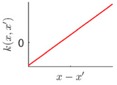	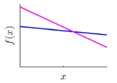
SE	Infinitely differentiable, offering smooth variations with a typical length scale	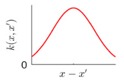	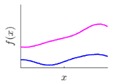
PER	With arbitrary roughness and period, suitable for periodic shape	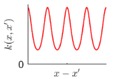	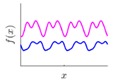
MC	Finite times differentiable, suitable for different roughness with appropriate parameters	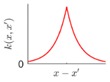	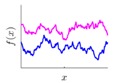
RQ	A mixture of SE with different length scales, more flexible with relatively more hyperparameters, suitable for smooth and multi-scaled shape	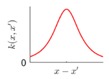	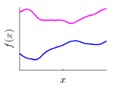
NN	Rapid or large variations with non-stationary spatial correlation, suitable for the irregular surfaces with random features, such as the complex terrain	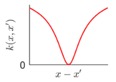	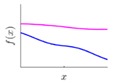
PP	Finite continuously differentiable, suitable for large continuous or fast-changing shape	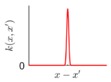	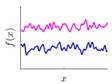

**Table 2:** GP modelling of various complex surfaces.

Designed Complex Surfaces	Covariance Kernel Functions	Residual Maps
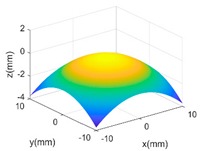	SE	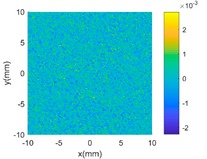
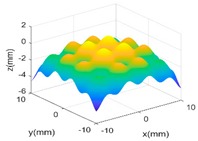	SE + PER	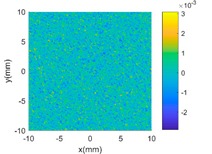
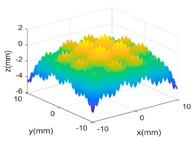	SE + PER + MC	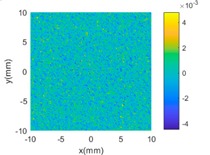
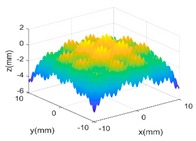	SE + PER + PER	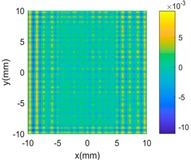

**Table 3:** A summary of the actual measurement result.

Measurement Strategy	Number of Points	RMS (μm)
Trigger probe dense measurement	6456	5.9
Laser scanner dense measurement	more than 40,000	11.2
Trigger probe adaptive measurement	493	5.7
Multi-sensor adaptive measurement	281	5.5
